# Identification of Specific Circular RNA Expression Patterns and MicroRNA Interaction Networks in Mesial Temporal Lobe Epilepsy

**DOI:** 10.3389/fgene.2020.564301

**Published:** 2020-09-25

**Authors:** Lachlan G. Gray, James D. Mills, Ashton Curry-Hyde, Sasha Devore, Daniel Friedman, Maria Thom, Catherine Scott, Roland D. Thijs, Eleonora Aronica, Orrin Devinsky, Michael Janitz

**Affiliations:** ^1^School of Biotechnology and Biomolecular Sciences, University of New South Wales Sydney, Sydney, NSW, Australia; ^2^Amsterdam UMC, Department of (Neuro)Pathology, Amsterdam Neuroscience, University of Amsterdam, Amsterdam, Netherlands; ^3^Department of Clinical and Experimental Epilepsy, Queen Square Institute of Neurology, University College London, London, United Kingdom; ^4^Centre for Medical Image Computing, University College London Institute of Neurology, London, United Kingdom; ^5^Stichting Epilepsie Instellingen Nederland, Heemstede, Netherlands; ^6^Comprehensive Epilepsy Center, New York University Langone Medical Center, New York, NY, United States; ^7^Paul Flechsig Institute for Brain Research, University of Leipzig, Leipzig, Germany

**Keywords:** circular RNAs, microRNAs, epilepsy, mesial temporal lobe epilepsy, RNA-Seq, gene expression

## Abstract

Circular RNAs (circRNAs) regulate mRNA translation by binding to microRNAs (miRNAs), and their expression is altered in diverse disorders, including cancer, cardiovascular disease, and Parkinson’s disease. Here, we compare circRNA expression patterns in the temporal cortex and hippocampus of patients with pharmacoresistant mesial temporal lobe epilepsy (MTLE) and healthy controls. Nine circRNAs showed significant differential expression, including circRNA-*HOMER1*, which is expressed in synapses. Further, we identified miRNA binding sites within the sequences of differentially expressed (DE) circRNAs; expression levels of mRNAs correlated with changes in complementary miRNAs. Gene set enrichment analysis of mRNA targets revealed functions in heterocyclic compound binding, regulation of transcription, and signal transduction, which maintain the structure and function of hippocampal neurons. The circRNA–miRNA–mRNA interaction networks illuminate the molecular changes in MTLE, which may be pathogenic or an effect of the disease or treatments and suggests that DE circRNAs and associated miRNAs may be novel therapeutic targets.

## Introduction

Circular RNAs (circRNAs) are a unique class of non-coding RNA found in human tissues and are linked to diverse diseases (Chen et al., 2016, 2018). CircRNAs originate from backsplicing of the precursor-mRNA (pre-mRNA) transcript. This alternative (back) splicing of pre-mRNA transcripts occurs when the spliceosome covalently joins the 3′ end of a downstream exon to the 5′ end of an upstream exon. Thus, circRNAs are covalently closed and lack polyadenylated 3′-tails or 5′-caps, making them exonuclease resistant and longer lived (∼48 h) than linear RNA (∼10 h; Curry-Hyde et al., 2020). The stability of circRNAs allows for packaging in extracellular vesicles, suggesting a role in cell-to-cell communication (Lasda and Parker, 2016). circRNAs may also act as microRNA (miRNA) sponges; that is, some circRNA sequences are complementary to miRNAs and may sequester these miRNAs, preventing them from binding to target mRNA (Hansen et al., 2013; Jeck et al., 2013).

Mesial temporal lobe epilepsy (MTLE) is a common human epilepsy and is often accompanied by hippocampal sclerosis (HS; Engel, 2001): a pathology associated with pharmacoresistance (Blümcke et al., 2013). HS is characterized by neuronal loss and gliosis, granule cell dispersion, and aberrant mossy fiber sprouting (axonal projection) maximal in the CA1 and dentate gyrus. The hippocampus is critical for short-term memory (Bauman et al., 2019). MTLE patients with HS (MTLE-HS), especially in the dominant (or both) hemisphere(s), exhibit memory deficits. Pharmacoresistant epilepsy and memory impairment may result from cell loss, synaptic reorganization, altered excitatory–inhibitory balance, and aberrant hippocampal discharges (Scharfman et al., 2003; Blümcke et al., 2009; Gelinas et al., 2016). Many MTLE patients undergo surgical resection if seizures are pharmacoresistant, with seizure-free rates of 60–80%, and reduced seizures in 95%. However, many surgical candidates do not undergo epilepsy surgery due to limited health care resources or fears of complications (Kang et al., 2016).

We recently reported linear RNA and small RNA transcriptome analysis in the temporal cortex and hippocampus of MTLE individuals. We found large transcriptomic changes for linear RNA in the hippocampus but not in the cortex. By contrast, differentially expressed (DE) small RNAs (e.g., miRNAs) were identified across both brain regions (Mills et al., 2020). Two other studies compare circRNAs in MTLE patients and controls, although brain regions from controls did not precisely match MTLE patients. The first, a microarray analysis, sampled a 5639 probe circRNA chip for human circRNA splicing sites. A > 3-fold DE was identified for 586 circRNAs; the top 10 dysregulated circRNAs were validated with real-time PCR (Gong et al., 2018). The second study found 254 DE down-regulated circRNAs and 188 DE up-regulated circRNAs (Li et al., 2018). These results may reflect tissue-specific differences in circRNA expression owing to the different source of tissues in MTLE patients versus controls.

Here, we investigated circRNA expression in the temporal cortex and hippocampus of MTLE and healthy individuals. Our results identify aberrant circRNA expression in MTLE individuals. We hypothesize that altered circRNA expression perturbs gene networks in MTLE by sequestering miRNAs from their target genes. Using bioinformatic techniques, we analyzed the miRNA sponging potential of DE circRNAs and assessed altered gene networks. To our knowledge, this is the first study to combine linear, circular, and miRNA transcriptome profiles in assessing MTLE molecular signals.

## Materials and Methods

### Data Acquisition

Our patient and control cohorts, tissue sampling, and RNA sequencing and analysis methodologies were reported in a previous study (Mills et al., 2020). This data set contains 14 postmortem control samples (6 temporal cortex and 8 hippocampal) and 24 surgical MTLE samples (7 cortical and 17 hippocampal). Demographic and clinical characteristics for each patient and control are summarized in Supplementary Table 1. Control tissues were obtained during the autopsy of age-matched individuals without neurological disease. The MTLE tissues were obtained at surgical resection. For total and small RNA-seq library preparations, ribosomal RNA (rRNA) was depleted, and paired-end sequencing was performed using HiSeq 4000 with read lengths of 151 nucleotides (nts) to a depth of 50 million and 20 million reads, respectively (Mills et al., 2020).

### Read Quality Analysis

To assess the quality of sequencing, each forward and reverse read file was analyzed with FastQC (Andrews, 2018; version 0.11.8). Trimmomatic (Bolger et al., 2014; version 0.36) was used to remove low-quality reads and sequencing adaptors from each read. Trimmomatic was run in paired-end mode with a phred33 score. Low-quality leading and trailing reads of 3 nt were removed with a sliding window of 4:15 and a minimum length of 30 nt. After trimming, FastQC determined the quality of trimmed reads. The small RNA data sets were assessed with FastQC and sequencing reads trimmed with Trimmomatic, which was run in paired-end mode with a phred33 score. Low-quality leading and trailing reads of 3 nt were removed with a sliding window of 4:15 and minimum length of 17 nt.

### Read Alignment

Sequencing reads were aligned to the human reference genome GRCh38 accessed from UCSC (Kent et al., 2002). Reads were mapped with STAR (Dobin et al., 2013; version 2.6.1b) to rapidly generate linear and chimeric reads and information pertaining to the uniquely mapped read quantity.

### Linear Detection

To analyze linear and small RNA transcripts, the indexed.bam file generated by STAR was parsed to StringTie (Pertea et al., 2015; version 1.3.4d) with the GRCh38 Ensembl or miRbase annotation.gtf for the small RNA data sets. StringTie was run with the -e flag to identify an abundance of known linear transcripts and ignore novel transcripts. The StringTie Python script prepDE.py created a matrix file of each gene and its respective expression across all samples.

### CircRNA Detection

We used the CIRCexplorer2 (Zhang et al., 2016; version 2.3.6) and DCC (Cheng et al., 2016; version 0.4.8) programs to detect circRNAs. Each program provides different degrees of functionality. For example, the output of CIRCexplorer2 provides information regarding the coding strand and how many exons compose a circRNA. Using DCC, we removed potential circRNAs with <2 reads and that were not expressed in at least 10 samples to reduce false positives.

With a custom Python script, the output files of these two programs were merged and filtered for counts per million mapped reads (CPM) values of ≥0.1. CPM normalizes circRNA abundance to account for the differences in the number of uniquely mapped reads across the samples. This filtration guarantees that the circRNAs detected by both tools are highly expressed and deemed as bona fide.

### Analysis of Expression Distribution

To determine whether detected circRNAs and linear transcripts cluster with respect to tissue or disease type, we performed a t-distributed stochastic neighbor embedding (t-SNE) machine learning algorithm with the R (R Development Core Team, 2013) package Rtsne (Van Der Maaten et al., 2014).

### Differential Expression Analysis

For the DE analysis, we used the R program edgeR (Robinson et al., 2009; Law et al., 2018). Two DE experiments were performed in which the control-cortex was compared to the MTLE-cortex and the control-hippocampus was compared to the MTLE-hippocampus. A circRNA or mRNA was removed if its read count was zero across ≥10 samples. DE analysis was carried out using the edgeR decideTests function; genes with a Benjamini–Hochberg adjusted *p*-value < 0.05 were considered statistically significant.

### miRNA Response Elements Analysis

To identify miRNA response elements (MREs) within the DE circRNAs, the ENCORI (Li et al., 2014; Dori and Bicciato, 2019) Argonaut (AGO)-CLIP-seq database was queried. A custom Python script searched for miRNA binding sites located within the specific circRNA transcript coordinates. Using a curl command to access the ENCORI API, we collected the miRNA–mRNA interaction data for all miRNAs binding to each circRNA. Within this command, we filtered the output for ≥1 supporting CLIP-seq experiments and ≥2 miRNA target-predicting programs. A custom Python script collected the read coverage determined by StringTie for each target gene’s transcript for each sample.

We employed edgeR to identify DE transcript sets for circRNAs of interest. For example, if the circRNA was DE in the hippocampus, we performed a linear DE of the target genes of the miRNAs predicted to bind that circRNA between the control-hippocampus and the MTLE-hippocampus. The DE matrix was input to ggplot (Wickham, 2006), ggrepel (Slowikowski, 2017), and dplyr (Wickham et al., 2017) to generate volcano plots to visualize DE patterns. To convert ensemble transcript identifiers to more readable gene symbols, we used the biomaRt (Durinck et al., 2009) R package.

### Gene Set Enrichment Analysis

To investigate the significance of DE linear transcripts, we utilized the SetRank (Simillion et al., 2017) GSEA algorithm. This algorithm increases the confidence of a gene ontology (GO) analysis by removing gene sets that are flagged as significant if their significance is due to an overlap with other gene sets. SetRank analyzed the tables of top-ranked genes generated by edgeR against a background gene set filtered for all GO databases, including biological processes, molecular function, cellular compartment, and REACTOME.

## Results

### Read Mapping and CircRNA Detection

The average percentage of uniquely mapped reads for each sample was 90%, illustrating the high quality of RNA template preparation and sequencing. CIRCexplorer2 detected significantly more circRNAs than DCC owing to DCC’s high stringency and low false-positive rate. After merging the circRNAs to find those commonly detected by both tools, 1515 circRNAs were detected across all samples; the quantity of circRNA was expected, as circRNA is highly abundant in the brain (Gokool et al., 2020). All uniquely mapped read values and circRNAs detected by CIRCexplorer2 and DCC and those detected by both tools are provided in Supplementary Table 1.

### t-SNE Analysis

**FIGURE 1:**
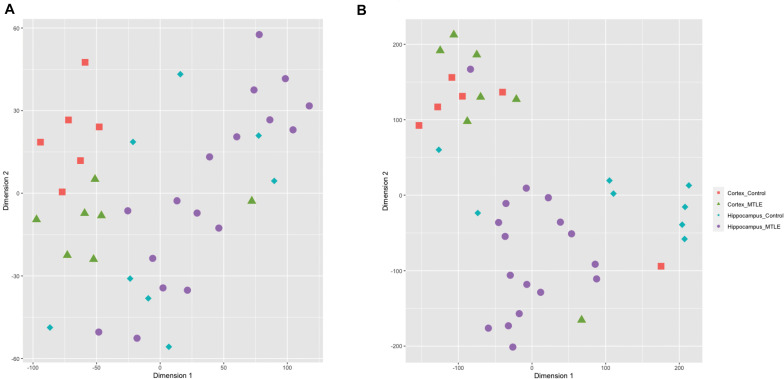
**(A)** t-SNE clustering of detected circRNAs. CircRNAs from the same tissue cluster together regardless of disease state. **(B)** t-SNE clustering of linear transcripts. Cortex samples cluster together, and hippocampal samples show more disorganized clustering.

To assess sample clustering, we employed the R package Rtsne to perform a t-SNE machine learning analysis on the detected circRNA expression levels for each sample (Figure 1A). CircRNA expression was clustered by brain region rather than disease versus control, suggesting region-specific functionality of circRNAs. A t-SNE analysis of detected linear transcripts revealed less organized clustering of samples (Figure 1B). Cortex samples were clustered closely together, and hippocampus samples were less clustered.

### CircRNA Differential Expression in MTLE

Two separate differential gene expression comparisons were performed: control-cortex versus MTLE-cortex and control-hippocampus versus MTLE-hippocampus. Within the cortex samples, there was a single DE circRNA that is derived from the calmodulin regulated spectrin associated protein 1 (*CAMSAP1*) gene. Comparisons between MTLE and control hippocampal tissue revealed eight DE circRNAs. Table 1 summarizes the metrics of the nine DE circRNAs in both tissues.

**TABLE 1:** List of DE circRNA in MTLE cortex and hippocampus.

CircRNA ID—GRCh38*	Gene ID	Gene Descriptions	Strand	LogFC**	AveExp (CPM)***	*p*-value	Adjusted *p*-value
chr9:135866456–135883078	*CAMSAP1*	Calmodulin Regulated Spectrin Associated Protein 1	–	+ 3.78	-4.309	2.85E-06	0.011
chr3:161224930–161235212	*NMD3*	*NMD3* Ribosome Export Adaptor	+	+ 3.37	–3.5	3.18E-06	0.008
chr8:26391243–26408376	*BNIP3L*	BCL2 Interacting Protein 3 Like	+	+ 2.63	–4.63	4.56E-06	0.008
chr6:98899268–98934879	*FBXL4*	F-Box and Leucine Rich Repeat Protein 4	–	+ 2.68	–4.59	8.57E-06	0.01
chr4:165989381–166025431	*TLL1*	Tolloid Like 1	+	+ 2.56	–4.71	2.37E-05	0.019
chr2:220630379–220634128	*AC067956.1*	Long non-coding RNA SLC4A3-8	+	–2.95	–4.49	2.64E-05	0.019
chr1:213077696–213129889	*RPS6KC1*	Ribosomal Protein S6 Kinase C1	+	+ 2.43	–4.8	3.29E-05	0.02
chr11:113202379–113214511	*NCAM1*	Neural Cell Adhesion Molecule 1	+	+ 2.3	–4.88	6.65E-05	0.03
chr5:79439010–79457018	*HOMER1*	Homer Scaffold Protein 1	–	–0.61	1.3	5.22E-05	0.03

*Values underlined indicate cortex-specific circRNA.**FC: fold change in reference to healthy control.***AveExp: average expression.

**FIGURE 2:**
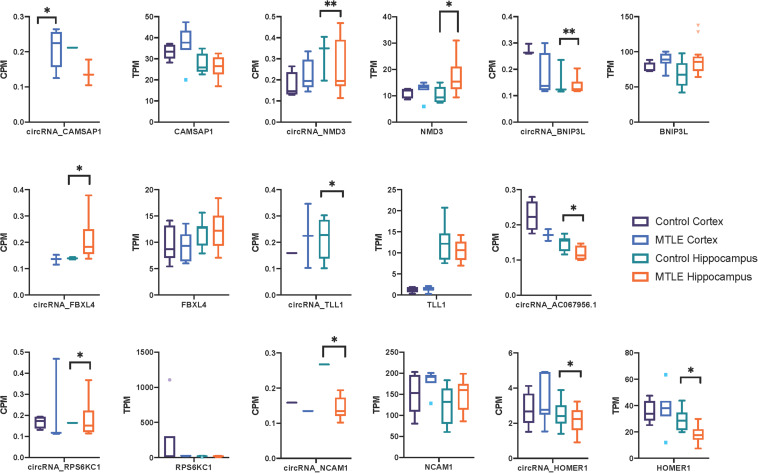
Box plots of CPM and TPM values for the nine differentially expressed circRNAs and linear transcripts across the two conditions in the cortex and hippocampus. CircRNA and parent gene expression do not correlate, which suggests that these circRNAs are not dependent on the linear expression of their host genes. No linear transcripts were detected for *AC067956.1*. ^*^<0.05 and ^**^<0.01.

Next, we investigated expression patterns of linear transcripts expressed by genes co-expressing DE circRNAs. Figure 2 shows the distribution of circRNA and linear transcript expression levels within individual biological replicates. Only two genes (Figure 2), Homer protein homolog 1 (*HOMER1*), and *NMD3* ribosome export adaptor (*NMD3*), revealed significant DE of linear and circular products between MTLE and control hippocampus.

### Estimation of miRNA Binding Capacity by DE CircRNAs

The ENCORI database provides AGO-CLIPseq data for circRNAs with miRNA-AGO complex binding sites. Because ENCORI uses the GRCh37 coordinates, each DE circRNA backspliced junction (BSJ) coordinate was converted to GRCh37 using the NCBI remap tool^1^. Using a custom Python script, we identified miRNA binding within the start and end coordinates, defining the BSJ of each DE circRNA. All DE circRNAs, except neural cell adhesion molecule 1 (*NCAM1*) and *AC067956.1*, exhibited miRNA binding potential (Table 2). The miRNAs with binding sites on each circRNA and their binding locations as GRCh38 coordinates are included in Supplementary Table 2.

**TABLE 2:** miRNA binding capacity to DE circRNAs.

CircRNA ID—GRCh37	Gene ID	No. of miRNAs bound
chr9:138758302–138774924	*CAMSAP1*	11
chr3:160942718–160953000	*NMD3*	15
chr8:26248759–26265892	*BNIP3L*	15
chr6:99347144–99382755	*FBXL4*	57
chr4:166910533–166946583	*TLL1*	7
chr2:221495100–221498849	*AC067956.1*	0
chr1:213251038–213303232	*RPS6KC1*	11
chr11:113073101–11308523	*NCAM1*	0
chr5:78734833–78752841	*HOMER1*	12

### CircRNA–miRNA–mRNA Interaction Analysis

**FIGURE 3:**
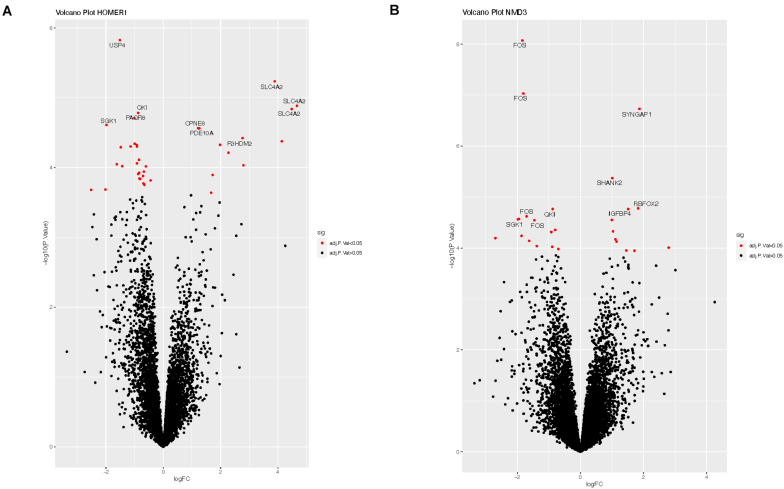
**(A)** Volcano plot of the linear transcripts that are targeted by the miRNAs (see Table 2), which were predicted to interact with circRNA-*HOMER1*. **(B)** Volcano plot of the linear transcripts that are targeted by the miRNAs, which were predicted to interact with circRNA-*NMD3.* The X- and Y-axes of this plot show the log fold change and -log_10_ adjusted *p*-value, respectively, indicating how differentially expressed a transcript is when compared to controls. Red indicates the transcripts with adjusted *p*-values of <0.05, which suggests that these linear transcripts were affected by this set of miRNAs interacting with circRNA. Labeled transcripts are the top 10 most DE.

We explored potential associations between DE circRNAs, the miRNAs predicted to bind these circRNAs, and linear RNA targets that interact with these miRNAs. For example, circRNA-*HOMER1* was DE in the hippocampus, so DE analysis of mRNAs, targeted by the 12 miRNAs predicted to bind to circRNA-*HOMER1* (Table 2), was conducted on hippocampus samples. The edgeR-generated adjusted *p*-values were plotted to visualize transcript distribution as a volcano plot (Figure 3A). For circRNA-*HOMER1*, of the 7629 total linear transcripts, 37 linear transcripts were DE with 12 up-regulated and 25 down-regulated in the MTLE samples. A DE of the targets of the 15 miRNAs with binding sites on circRNA-NMD3 revealed that, of the 10,830 total linear transcripts, 27 were DE with 11 up-regulated and 16 down-regulated in the hippocampus (Figure 3B). All volcano plots for linear transcripts interacting with miRNAs are shown in Supplementary Figures 1A–G.

### Gene Set Enrichment Analysis

For the transcripts targeted by bound miRNAs, a GO analysis was performed with SetRank, a GSEA algorithm that removes false-positive hits. By utilizing detailed significance tests and graph theory, SetRank addresses the gene overlap and multiple testing problems associated with most GSEA algorithms and allows for querying of multiple different databases simultaneously (Simillion et al., 2017).

**FIGURE 4:**
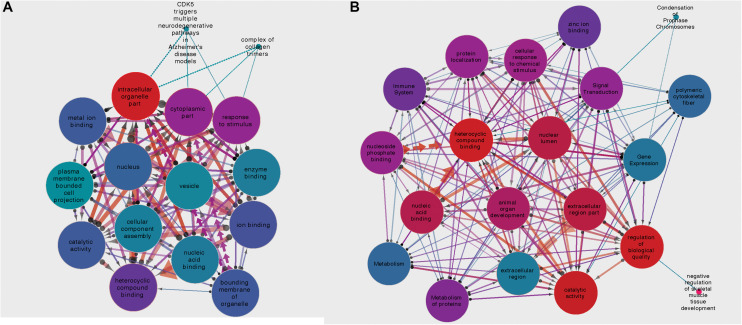
**(A)** Gene set interaction network for the miRNA targets composing the HOMER1 circRNA–miRNA–mRNA interaction network. **(B)** Gene set interaction network for the miRNA targets composing the NMD3 circRNA–miRNA–mRNA interaction network. Node color indicates the corrected *p*-value, edge thickness relates to the size of intersection between two gene sets, and edge direction points from the least significant to the most significant.

For the circRNA-*HOMER1* interaction network, the proteins were revealed to be located in the intracellular organelle, cytoplasm, and nucleus cellular components and had the molecular functions of heterocyclic compound binding and biological process response to stimulus. For circRNA-*NMD3*, the proteins were attributed to the nuclear lumen cellular compartment and had the molecular functions of heterocyclic compound binding and catalytic activity. Gene set interaction networks for HOMER1 and NMD3 generated by SetRank and visualized within Cytoscape (Su et al., 2014) are shown in Figure 4 and represent how each of the GSEA terms relate to each other. Nodes are color-coded by their corrected *p*-values, and the thickness and direction of the edges represent the size of the intersection and points from the least to the most significant. A full list of enriched terms ranked in order of importance for each DE circRNA–miRNA–mRNA network is included in Supplementary Table 3. Additionally, SetRank-generated GSEA membership files reveal to which GSEA terms each protein is attributed, and these are included as Supplementary Files 1A–F.

## Discussion

In this study, we present, for the first time, changes in the circular transcriptome landscape within the temporal cortex and hippocampus of MTLE patients. An integrated bioinformatics analysis revealed circRNA–miRNA–mRNA regulatory networks that are specifically formed in MTLE brains, thus showing that multiple compartments of the transcriptome are perturbed in this type of epilepsy. Among the thousands of neuronally expressed circRNAs, we identified one DE circRNA in the cortex and eight DE in the hippocampus of MTLE patients. Both linear and circular transcripts for two genes, *NMD3* and *HOMER1*, were DE in the hippocampus. The concurrent expression pattern suggests that these circRNAs may be co-expressed with linear transcripts. Alternatively, these circRNAs may be regulating their own transcription through interactions with U1 small nuclear ribonucleoprotein (snRNP) or by regulating RNA polymerase II-mediated transcription (Kristensen et al., 2019).

We compared our detected circRNAs to the list of circRNAs detected by Li et al. (2018) and found overlapping circRNAs, which showed a similar direction of expression. For circRNAs detected in the cortex, there was an overlap of 23 circRNAs and, in hippocampus, an overlap of 26 circRNAs. None of our DE circRNAs were detected. The lack of complete overlap may be explained by differences in bioinformatic analysis and the lack of tissue specificity in the aforementioned study.

Our bioinformatics pipeline established that seven of the nine DE circRNAs could be classified as miRNA sponges, i.e., they contained sequences complementary to miRNAs. Our interaction network analysis determined the effects of circRNA expression relative to miRNA binding to target mRNAs. These DE target mRNAs revealed functions in signal transduction and transcription for neurons of the hippocampus, implying a potential role in the etiology of MTLE-HS.

For circRNA-*NMD3* and the set of miRNAs binding to circRNA-*FBXL4*, there was an overlap of miRNAs from the same family (Supplementary Table 2). This may reflect sequence conservation across miRNAs of the same family because miRNA families often share sequence, structure, and function (Kaczkowski et al., 2009).

CircRNA-*NMD3* is expressed from the *NMD3* ribosome export adaptor gene locus. *NMD3* encodes a protein that transports the ribosomal 60S subunit to the cytoplasm (Trotta et al., 2003). In MTLE, the dentate gyrus is where excitatory mossy fiber axons lose contact with mossy cells and migrate back into the molecular layer as the densely packed granule cells migrate and disperse (Schmeiser et al., 2017). CircRNA-*NMD3* is shown to be DE in the hippocampus of MTLE patients. CircRNA-*NMD3* interacts with 15 miRNAs, and further investigation of those miRNAs identified DE of their target linear transcripts. Of the 27 DE transcripts, 11 were up-regulated and 16 were down-regulated in the hippocampus. GSEA of those proteins revealed involvement in molecular function, cellular component, biological processes, and REACTOME pathways. The first ranked term, heterocyclic compound binding, might be of significance as these compounds have been used in multiple antiepileptic drugs (Wei et al., 2015). These DE proteins in the hippocampus of MTLE individuals may inhibit the binding of these compounds and may explain the high rates of pharmacoresistance associated with the disease. REACTOME pathways for these proteins include signal transduction and metabolism of proteins, which may be affecting synaptic stimulation. As similarly revealed by Mills et al. (2020), the DE transcripts are shown to have a function in the immune system.

CircRNA-*FBXL4* is expressed from the gene locus of the F-Box and leucine-rich repeat protein 4. The *FBXL4* protein colocalizes with mitochondria to maintain mtDNA (Bonnen et al., 2013). CircRNA-*FBXL4* exhibits binding sites for 57 miRNAs of which seven comprise the miRNA family hsa-miR-378. There were 83 DE transcripts, and 33 were up-regulated and 50 down-regulated. GSEA revealed that the proteins involved in this network have biological processes of protein localization and regulation of the protein modification process. REACTOME terms included gene expression and metabolism of proteins. The cellular compartment location of these proteins is shown to be in the endoplasmic reticulum, which has a clear relation to these protein-specific functions. The unspecific functions of these proteins may be due to the enrichment for miRNA binding sites on circRNA-*FBXL4* and the subsequently large pool of mRNA targets.

We observed significant differences in the expression levels of circRNA-*HOMER1* and linear transcripts between MTLE hippocampus and controls. The *HOMER1* gene encodes proteins involved in neuronal activity that bridge Group I metabotopic glutamate receptors (mGluR1/5) with inositol 1,4,5-triphosphate receptors (IP3Rs) on the endoplasmic reticulum, synaptic calcium ion channels, and *NMDA* receptor signaling complexes (Aloisi et al., 2017). After synaptic activity, a short splice variant *HOMER1a* is expressed as an immediate early gene and may antagonize group I mGluR activation (Tappe and Kuner, 2006). *HOMER1a* is implicated in epileptogenesis (Potschka et al., 2002; Celikel, 2007; Wagner et al., 2013). CircRNA-*HOMER1* has binding sites for 12 miRNAs. There were 37 transcripts shown to be DE with 12 up-regulated and 25 down-regulated. GSEA of these proteins similarly revealed a molecular function in heterocyclic compound binding and ion binding. This may have the effect of modifying the hippocampal neurons’ ability to bind heterocyclic neurotransmitters and antiepileptic medications and maintain the ion exchange required for generating action potentials.

Our study identified nine DE circRNAs between the cortex and hippocampus of individuals with MTLE. Of these, seven exhibited miRNA sponging characteristics, which likely have phenotypic roles in MTLE by contributing to, or resulting from, HS. Dissecting the effects of each miRNA to these interaction networks can advance our understanding of the individual roles played by miRNA sponging in MTLE pathogenesis and identify targets for further research. Future studies should investigate the effects of knocking down these target circRNAs in neuronal cell lines using *in vivo* circRNA-specific knockdown (Zimmerman et al., 2020) and assessing the subsequent changes in miRNA activity. If unbound miRNAs regulate mRNA translation, quantitative changes in protein expression should result. Additionally, more experimental validations are required to define the mechanisms of circRNA–miRNA binding and how the secondary RNA structure of a circRNA can facilitate these interactions. For circRNAs that do not interact with miRNAs, other mechanisms of action should be explored, such as RBP binding and protein scaffolding.

## Data Availability Statement

Publicly available datasets were analyzed in this study. This data can be found here: European Genome-phenome Archive (EGA) which is hosted by the EBI and the CRG, under the accession number: EGAS00001003922.

## Author Contributions

MJ, EA, and OD conceived of the study. JM, SD, DF, MT, CS, and RT contributed to primary clinical and analytical design of the study. LG performed the data analysis. AC-H assisted with the data analysis. LG, JM, EA, OD, and MJ wrote the manuscript. AC-H, SD, and DF provided feedback to the manuscript. All authors read and approved the final manuscript.

## Conflict of Interest

The authors declare that the research was conducted in the absence of any commercial or financial relationships that could be construed as a potential conflict of interest.

## Footnotes

1 https://www.ncbi.nlm.nih.gov/genome/tools/remap

## Supplementary Material

Supplementary Figure 1 Volcano plots showing DE of transcripts the targeted by miRNA predicted to interact with a circRNA. **(A)** circRNA-*BNIP3L*. **(B)** circRNA-*CAMSAP1*. **(C)** circRNA-*FBXL4*. **(D)** circRNA-*HOMER1*. **(E)** circRNA-*NMD3*. **(F)** circRNA-*RPS6KC1*. **(G)** circRNA-*TLL1.*

Supplementary Table 1 Clinical details of each tissue sample file indicating whether an individual is in control or disease group and the brain region. Details on the percentage of uniquely mapped reads and the total circRNAs detected by either CIRCexplorer2 or DCC and those commonly detected by both tools are also provided.

Supplementary Table 2 The miRNAs and their binding sites as GRCh38 coordinates for each circRNA.

Supplementary Table 3 GSEA terms generated by SetRank for the linear targets of miRNAs with binding sites on circRNAs. The SetRank is a score of significance as each term is ranked on its importance.

Supplementary File 1 SetRank membership files showing to which GSEA terms each gene is attributed. **(A)** *BNIP3l*. **(B)** *FBXL4*. **(C)** *HOMER1*. **(D)** *NMD3*. **(E)** *RPS6KC1*. **(F)** *TLL1*.

## References

[B1] Aloisi E. Le Corf K. Dupuis J. Zhang P. Ginger M. Labrousse V. (2017). Altered surface mGluR5 dynamics provoke synaptic NMDAR dysfunction and cognitive defects in Fmr1 knockout mice. *Nat. Commun.* 8 1–14. 10.1038/s41467-017-011911192 29062097 PMC5653653

[B2] Andrews S. (2018). FastQC a quality control tool for high throughput sequence data. *Babraham. Bioinfo.* 2018 3–5.

[B3] Bauman K. Devinsky O. Liu A. A. (2019). Temporal lobe surgery and memory: Lessons, risks, and opportunities. *Epil. Behav.* 101:106596. 10.1016/j.yebeh.2019.106596 31711868 PMC6885125

[B4] Blümcke I. Kistner I. Clusmann H. Schramm J. Becker A. J. Elger C. E. (2009). Towards a clinico-pathological classification of granule cell dispersion in human mesial temporal lobe epilepsies. *Acta Neuropathol.* 117 535–544. 10.1007/s00401-009-0512515 19277686

[B5] Blümcke I. Thom M. Aronica E. Armstrong D. D. Bartolomei F. Bernasconi A. (2013). International consensus classification of hippocampal sclerosis in temporal lobe epilepsy: A Task Force report from the ILAE Commission on Diagnostic Methods. *Epilepsia* 54 1315–1329. 10.1111/epi.12220 23692496

[B6] Bolger A. M. Lohse M. Usadel B. (2014). Trimmomatic: A flexible trimmer for Illumina sequence data. *Bioinformatics* 30 2114–2120. 10.1093/bioinformatics/btu170 24695404 PMC4103590

[B7] Bonnen P. E. Yarham J. W. Besse A. Wu P. Faqeih E. A. Al-Asmari A. M. (2013). Mutations in FBXL4 cause mitochondrial encephalopathy and a disorder of mitochondrial DNA maintenance. *Am. J. Hum. Genet.* 93 471–481. 10.1016/j.ajhg.2013.07.017 23993193 PMC3769921

[B8] Celikel T. (2007). Select overexpression of homer1a in dorsal hippocampus impairs spatial working memory. *Front. Neurosci.* 1:97–110. 10.3389/neuro.01.1.1.007.2007 18982121 PMC2518050

[B9] Chen B. J. Byrne F. L. Takenaka K. Modesitt S. C. Olzomer E. M. Mills J. D. (2018). Analysis of the circular RNA transcriptome in endometrial cancer. *Oncotarget* 9 5786–5796. 10.18632/oncotarget.23534 29464034 PMC5814174

[B10] Chen B. J. Mills J. D. Takenaka K. Bliim N. Halliday G. M. Janitz M. (2016). Characterization of circular RNAs landscape in multiple system atrophy brain. *J. Neurochem.* 139 485–496. 10.1111/jnc.13752 27470294

[B11] Cheng J. Metge F. Dieterich C. (2016). Specific identification and quantification of circular RNAs from sequencing data. *Bioinformatics* 32 1094–1096. 10.1093/bioinformatics/btv656 26556385

[B12] Curry-Hyde A. Ueberham U. Arendt T. Janitz M. (2020). Neural circular transcriptomes across mammalian species. *Genomics* 112 1162–1166. 10.1016/j.ygeno.2019.06.030 31255695

[B13] Dobin A. Davis C. A. Schlesinger F. Drenkow J. Zaleski C. Jha S. (2013). STAR: Ultrafast universal RNA-seq aligner. *Bioinformatics* 29 15–21. 10.1093/bioinformatics/bts635 23104886 PMC3530905

[B14] Dori M. Bicciato S. (2019). Integration of bioinformatic predictions and experimental data to identify circRNA-miRNA associations. *Genes* 10:642. 10.3390/genes10090642 31450634 PMC6769881

[B15] Durinck S. Spellman P. T. Birney E. Huber W. (2009). Mapping identifiers for the integration of genomic datasets with the R/Bioconductor package biomaRt. *Nat. Protoc.* 4 1184–1191. 10.1038/nprot.2009.97 19617889 PMC3159387

[B16] Engel J. Jr. (2001). Mesial temporal lobe epilepsy: What have we learned? *Neuroscientist* 7 340–352. 10.1177/107385840100700410 11488399

[B17] Gelinas J. N. Khodagholy D. Thesen T. Devinsky O. Buzsáki G. (2016). Interictal epileptiform discharges induce hippocampal-cortical coupling in temporal lobe epilepsy. *Nat. Med.* 22 641–648. 10.1038/nm.4084 27111281 PMC4899094

[B18] Gokool A. Anwar F. Voineagu I. (2020). The Landscape of Circular RNA Expression in the Human Brain. *Biol. Psych.* 87 294–304. 10.1016/j.biopsych.2019.07.029 31570194

[B19] Gong G. H. An F. M. Wang Y. Bian M. Wang D. Wei C. X. (2018). Comprehensive circular RNA profiling reveals the regulatory role of the CircRNA-0067835/miR-155 pathway in temporal lobe epilepsy. *Cell. Physiol. Biochem.* 51 1399–1409. 10.1159/000495589 30485839

[B20] Hansen T. B. Jensen T. I. Clausen B. H. Bramsen J. B. Finsen B. Damgaard C. K. (2013). Natural RNA circles function as efficient microRNA sponges. *Nature* 495 384–388. 10.1038/nature11993 23446346

[B21] Jeck W. R. Sorrentino J. A. Wang K. Slevin M. K. Burd C. E. Liu J. (2013). Circular RNAs are abundant, conserved, and associated with ALU repeats. *RNA* 19 141–157. 10.1261/rna.035667.112 23249747 PMC3543092

[B22] Kaczkowski B. Torarinsson E. Reiche K. Havgaard J. H. Stadler P. F. Gorodkin J. (2009). Structural profiles of human miRNA families from pairwise clustering. *Bioinformatics* 25 291–294. 10.1093/bioinformatics/btn628 19059941

[B23] Kang J. Y. Wu C. Tracy J. Lorenzo M. Evans J. Nei M. (2016). Laser interstitial thermal therapy for medically intractable mesial temporal lobe epilepsy. *Epilepsia* 57 325–334. 10.1111/epi.13284 26697969

[B24] Kent W. J. Sugnet C. W. Furey T. S. Roskin K. M. Pringle T. H. Zahler A. M. (2002). The Human Genome Browser at UCSC. *Genome Res.* 12 996–1006. 10.1101/gr.229102 12045153 PMC186604

[B25] Kristensen L. S. Andersen M. S. Stagsted L. V. W. Ebbesen K. K. Hansen T. B. Kjems J. (2019). The biogenesis, biology and characterization of circular RNAs. *Nat. Rev. Genet.* 20 675–691. 10.1038/s41576-019-0158157 31395983

[B26] Lasda E. Parker R. (2016). Circular RNAs co-precipitate with extracellular vesicles: A possible mechanism for circrna clearance. *PLoS One* 11 1–11. 10.1371/journal.pone.0148407 26848835 PMC4743949

[B27] Law C. W. Alhamdoosh M. Su S. Dong X. Tian L. Smyth G. K. (2018). RNA-seq analysis is easy as 1-2-3 with limma, Glimma and edgeR. *F1000Research* 5:1408. 10.12688/f1000research.9005.3 27441086 PMC4937821

[B28] Li J. Lin H. Sun Z. Kong G. Yan X. Wang Y. (2018). High-Throughput data of circular RNA profiles in human temporal cortex tissue reveals novel insights into temporal lobe epilepsy. *Cell. Physiol. Biochem.* 45 677–691. 10.1159/000487161 29428937

[B29] Li J. H. Liu S. Zhou H. Qu L. H. Yang J. H. (2014). StarBase v2.0*: Decoding miRNA-ceRNA, miRNA-ncRNA and protein-RNA interaction networks from large-scale CLIP-Seq data*. *Nucleic Acids Res.* 42 D92–D97. 10.1093/nar/gkt1248 24297251 PMC3964941

[B30] Mills J. D. van Vliet E. A. Chen B. J. Janitz M. Anink J. J. Baayen J. C. (2020). Coding and non-coding transcriptome of mesial temporal lobe epilepsy: Critical role of small non-coding RNAs. *Neurobiol. Dis.* 134:104612. 10.1016/j.nbd.2019.104612 31533065

[B31] Pertea M. Pertea G. M. Antonescu C. M. Chang T. C. Mendell J. T. Salzberg S. L. (2015). StringTie enables improved reconstruction of a transcriptome from RNA-seq reads. *Nat. Biotechnol.* 33 290–295. 10.1038/nbt.3122 25690850 PMC4643835

[B32] Potschka H. Krupp E. Ebert U. Gümbel C. Leichtlein C. Lorch B. (2002). Kindling-induced overexpression of Homer 1A and its functional implications for epileptogenesis. *Eur. J. Neurosci.* 16 2157–2165. 10.1046/j.1460-9568.2002.02265.x 12473083

[B33] R Development Core Team (2013). *A Language and Environment for Statistical Computing. R Found. Stat. Comput. 2.* Austria: R Development Core Team.

[B34] Robinson M. D. McCarthy D. J. Smyth G. K. (2009). edgeR: A Bioconductor package for differential expression analysis of digital gene expression data. *Bioinformatics* 26 139–140. 10.1093/bioinformatics/btp616 19910308 PMC2796818

[B35] Scharfman H. E. Sollas A. L. Berger R. E. Goodman J. H. (2003). Electrophysiological evidence of monosynaptic excitatory transmission between granule cells after seizure-induced mossy fiber sprouting. *J. Neurophysiol.* 90 2536–2547. 10.1152/jn.00251.2003 14534276

[B36] Schmeiser B. Zentner J. Prinz M. Brandt A. Freiman T. M. (2017). Extent of mossy fiber sprouting in patients with mesiotemporal lobe epilepsy correlates with neuronal cell loss and granule cell dispersion. *Epilepsy Res.* 129 51–58. 10.1016/j.eplepsyres.2016.11.011 27907826

[B37] Simillion C. Liechti R. Lischer H. E. L. Ioannidis V. Bruggmann R. (2017). Avoiding the pitfalls of gene set enrichment analysis with SetRank. *BMC Bioinformatics* 18:1–14. 10.1186/s12859-017-15711576 28259142 PMC5336655

[B38] Slowikowski K. (2017). *ggrepel: repulsive text and label geoms for “ggplot2”. R package version* 0.7.0. https://CRAN.R-project.org/package=ggrepel.

[B39] Su G. Morris J. H. Demchak B. Bader G. D. (2014). Biological Network Exploration with Cytoscape 3. *Curr. Protoc. Bioinforma.* 47 8–13. 10.1002/0471250953.bi0813s47 25199793 PMC4174321

[B40] Tappe A. Kuner R. (2006). Regulation of motor performance and striatal function by synaptic scaffolding proteins of the Homer1 family. *Proc. Natl. Acad. Sci. U. S. A.* 103 774–779. 10.1073/pnas.0505900103 16407107 PMC1325014

[B41] Trotta C. R. Lund E. Kahan L. Johnson A. W. Dahlberg J. E. (2003). Coordinated nuclear export of 60s ribosomal subunits and NMD3 in vertebrates. *EMBO J.* 22 2841–2851. 10.1093/emboj/cdg249 12773398 PMC156746

[B42] Van Der Maaten L. Courville A. Fergus R. Manning C. (2014). *Accelerating t-SNE using Tree-Based Algorithms.* Available online at: http://homepage.tudelft.nl/19j49/tsne, [accessed November 28, 2019]

[B43] Wagner K. V. Hartmann J. Mangold K. Wang X. D. Labermaier C. Liebl C. (2013). Homer1 mediates acute stress-induced cognitive deficits in the dorsal hippocampus. *J. Neurosci.* 33 3857–3864. 10.1523/JNEUROSCI.4333-12.2013 23447597 PMC6619309

[B44] Wei C. X. Bian M. Gong G. H. (2015). Current research on antiepileptic compounds. *Molecules* 20 20741–20776. 10.3390/molecules201119714 26610448 PMC6332177

[B45] Wickham H. (2006). *An introduction to ggplot: An implementation of the grammar of graphics in R*, (Netherland: Springer). 1–8.

[B46] Wickham H. Francois R. Henry L. Müller K. (2017). *Dplyr: a Grammar of Data Manipulation, 2013.* Available online at: https://github.com/hadley/dplyr. version 0.1.

[B47] Zhang X. O. Dong R. Zhang Y. Zhang J. L. Luo Z. Zhang J. (2016). Diverse alternative back-splicing and alternative splicing landscape of circular RNAs. *Genome Res.* 26 1277–1287. 10.1101/gr.202895.115 27365365 PMC5052039

[B48] Zimmerman A. J. Hafez A. K. Amoah S. K. Rodriguez B. A. Dell’Orco M. Lozano E. (2020). A psychiatric disease-related circular RNA controls synaptic gene expression and cognition. *Mol. Psychiatry* 27 1–16. 10.1038/s41380-020-0653-654 PMC7577899 31988434

