# Supplementary figures and images for: Identification of Specific Circular RNA Expression Patterns and MicroRNA Interaction Networks in Mesial Temporal Lobe Epilepsy
Source: Front Genet. 2020 Sep 25;11:564301. doi: 10.3389/fgene.2020.564301 (PMC7546880; doi:10.3389/fgene.2020.564301)

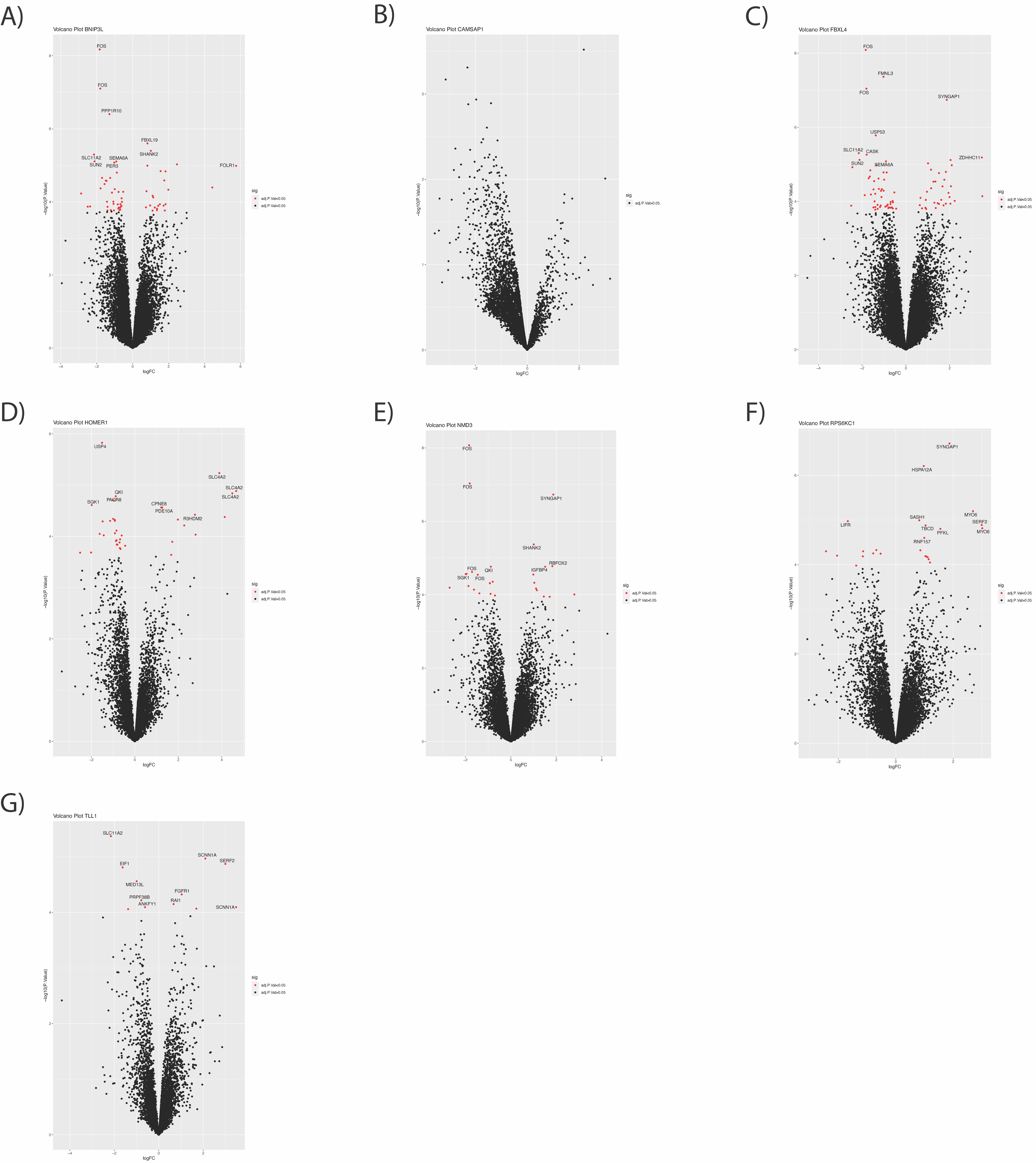

Supplement: Supplementary Figure 1 — Volcano plots showing DE of transcripts the targeted by miRNA predicted to interact with a circRNA. (A) circRNA-BNIP3L. (B) circRNA-CAMSAP1. (C) circRNA-FBXL4. (D) circRNA-HOMER1. (E) circRNA-NMD3. (F) circRNA-RPS6KC1. (G) circRNA-TLL1. [file Image_1.JPEG]
